# Examining the Pharmacist Labor Supply in the United States: Increasing Medication Use, Aging Society, and Evolution of Pharmacy Practice

**DOI:** 10.3390/pharmacy7030137

**Published:** 2019-09-19

**Authors:** Jonathan H. Watanabe

**Affiliations:** Division of Clinical Pharmacy, Skaggs School of Pharmacy and Pharmaceutical Sciences, University of California San Diego, La Jolla, CA 92093-0657, USA; jon.watanabe@outlook.com; Tel.: +1-858-822-1069

**Keywords:** pharmacist labor supply, pharmaceutical care, geriatrics, medication costs

## Abstract

The increasing number of pharmacists in the US has generated concern regarding potential oversupply. A 2018 analysis from the National Center for Health Workforce Analysis (NCHWA) in the US projected a best case scenario of an oversupply of more than 18,000 pharmacists in the year 2030. In this commentary, the limitations of this general health labor force analysis by the NCHWA are described. The goal of this work was to provide a more nuanced examination of the pharmacist labor demand in the US. Data from the US Bureau of Labor Statistics (BLS) and the US Medical Expenditure Panel Survey (MEPS) were utilized to examine, annually over a ten year period ending in 2017, the number of pharmacists, the ratio of pharmacists to persons living in the US, the ratio of pharmacists to older adults living in the US, and the ratio of medications to pharmacists. The number of pharmacists grew from 266,410 in 2008 to 309,330 in 2017. As anticipated, despite a growing US population, the ratio of people living in the US per pharmacist dropped unabated from 1141 to 1053 from 2008 to 2017, respectively. However, the reverse trend was observed for the ratio of persons 65 years or older per pharmacist. This ratio increased from 146.1 older adults to each pharmacist in 2008 to 164.3 in 2017. The accelerating demographic shift to an older population is also reversing an overall trend in the number of medications to pharmacist that will continue for the foreseeable future. While the ratio of medications to pharmacist dropped overall from 2008 to 2016, it has begun to rise again from 2016 to 2017. Beyond the increasing number of medications attributable to a rapidly aging population, there is a growing demand for clinical care from pharmacists due to the maturing environment of complex, costly medications for chronic disease treatment. As the portion of total health expenditure is increasingly devoted to medications and the US health delivery system continues its movement to community-based care, the demand for pharmacist care will require a larger number of pharmacists trained for advanced-practice care.

## 1. Introduction

Analysis of the pharmacist labor supply presents an interesting and, at times, paradoxical assessment of future demand. A rapidly aging population, coupled with increased usage of pharmaceuticals, has ushered in a new era of activities and demands for medication experts [1]. A recently published study by this author demonstrated the inordinate cost on the health care system, equaling $528.4 billion, attributable to morbidity and mortality due to non-optimized medication regimens. In that manuscript, a scale-up of comprehensive medication management (CMM) by clinical pharmacists to expand pharmaceutical care for patients is proposed as an evidence-based solution to reduce non-optimized medication regimens and the commensurate medical costs borne by society [2]. Currently, CMM occurs regularly in health care systems such as Kaiser Permanente and the Veterans Health Administration [3,4]. If CMM adoption expanded broadly to other settings, the increased demand for pharmacist labor would manifest rapidly. There are indications that this expansion is taking root. Recently proposed state legislation in California that would provide explicit CMM services to Medicaid enrollees has received bipartisan support, with endorsements from two state-wide physician organizations [5]. However, discussions regarding the future of the US pharmacy field are clouded by federal projections of general oversupply. The objectives of this commentary were to clarify limitations in the federal analysis, to perform a more pharmacist-specific analysis of labor supply using validated US government labor and health data, and to discuss implications and solutions to the growing reliance on pharmaceuticals societally.

On a US national level, updated projections of estimated future demand for health professionals participating in the delivery of medical-related services is performed by the National Center for Health Workforce Analysis (NCHWA) of the Health Resources and Services Administration (HRSA) of the US Department of Health and Human Services [6]. In the NCHWA, pharmacists’ labor supply and demand was most recently modeled in 2018 in the Allied Health Workforce Projections, a 2016–2030 report using the Health Workforce Simulation Model (HWSM) developed by HRSA. Importantly, the methods section of the NCHWA concedes the limitation that the basic framework of the HWSM remains the same across all professions, included in the category “Allied Health and Select Other Occupations”. Hence, the same model framework that is applied for health professions as disparate as audiologists, pharmacy aides, and clinical laboratory technicians, is applied for pharmacists [7]. Given the potential demand increase for pharmacists if direct patient-care services performed by pharmacists are augmented broadly, the limitation of employing the same basic model for pharmacists designed for health professions not expected to transform is a fundamental weakness. The HWSM produced two separate estimates of demand for pharmacists. The base case Scenario One assumes that 2016 health care use and delivery for pharmacist services will remain constant over the forecast period from 2016 to 2030. Scenario Two incorporates potential influence of population-level changes attributable to evolving health care system trends and goals. Examples of incorporation of population health changes analyzed in Scenario Two include assumptions of better control of diabetes or small decreases in excess body weight. The report again concedes explicitly that alterations in labor demand for pharmacists, due to innovations in health care delivery models, reform in payment structure, interdisciplinary-team care, patient behaviors relevant to health-seeking of pharmacists, and other system-level components, are difficult to account for in the HWSM. Scenario One estimated that total number of pharmacists would grow 36% by 2030 to 410,490 pharmacists in the US with an estimated demand in 2030 of 359,770. Scenario One translates to an oversupply of pharmacists of 50,720 in 2030. The Scenario Two analysis that attempted to incorporate an evolution of health system trends employed the same equation to arrive at the same pharmacist supply estimates in 2030 as Scenario One. Consequently, the estimated supply of pharmacists in 2030 would remain 410,490. In Scenario Two, demand was modified by the adjusted factors to yield an estimated demand of 391,850. This value represents an 8.9% increase in estimated demand, greater than Scenario One. However, it still translates to a predicted oversupply of pharmacists in 2030 of 18,640 [6]. As described earlier, while it is informative to have the crude estimates based on HWSM, the estimates are potentially biased by a general model structure that does not capture the material shift in practice of pharmacists as more move from a product-based profession to one of clinical and cognitive service delivery.

## 2. Examination of Bureau of Labor Statistics Data

**Figure 1:**
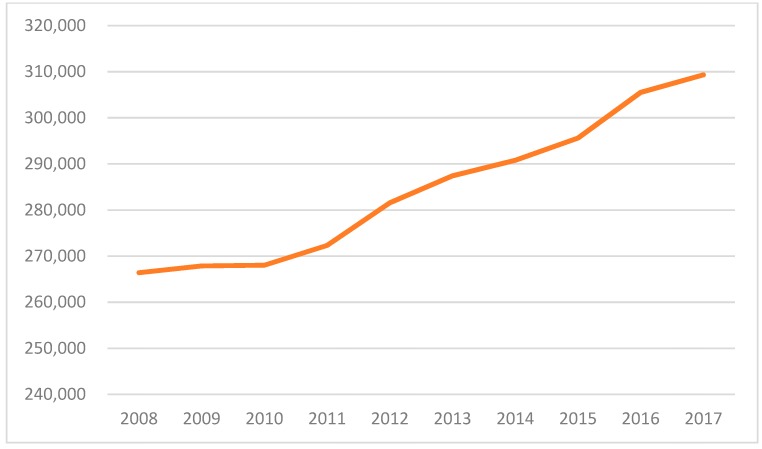
Number of pharmacists in the US (2008 to 2017).

**Figure 2:**
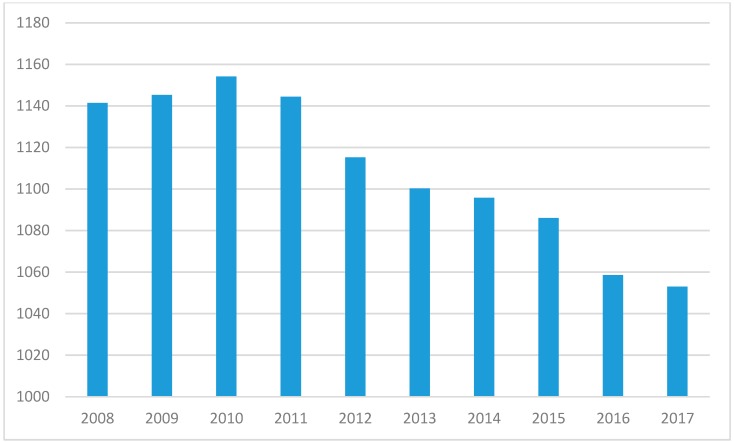
Ratio of persons in the US to pharmacist (2008 to 2017).

For the reasons given in the preceding section, it was important to complete a unique analysis that examined pharmacist labor using data from the US Bureau of Labor Statistics (BLS) and the most recent demographic and health trend data contained in the Medical Expenditure Panel Survey (MEPS), sponsored by the US Agency for Healthcare Research and Quality (AHRQ) [8,9]. To complete this, BLS statistics were downloaded for the most recent ten years of available data. Using the most recent MEPS data, average numbers of prescribed medications for the population under 65 years old and those 65 years and above were determined. Population changes and shifting composition of older adults in the population over time were also measured via MEPS data. Examining the past ten years of BLS data reveals a clear increase in the number of pharmacists propelled by an increase in the number of schools of pharmacy nationally [10]. While the US population continued to grow, the pace of pharmacist production was even greater, with the number of pharmacists increasing from 266,410 in 2008 to 309,330 in 2017 (Figure 1). This translated to the ratio of US residents per pharmacist dropping unabated from 1141 to 1053 from 2008 to 2017 (Figure 2).

**Figure 3:**
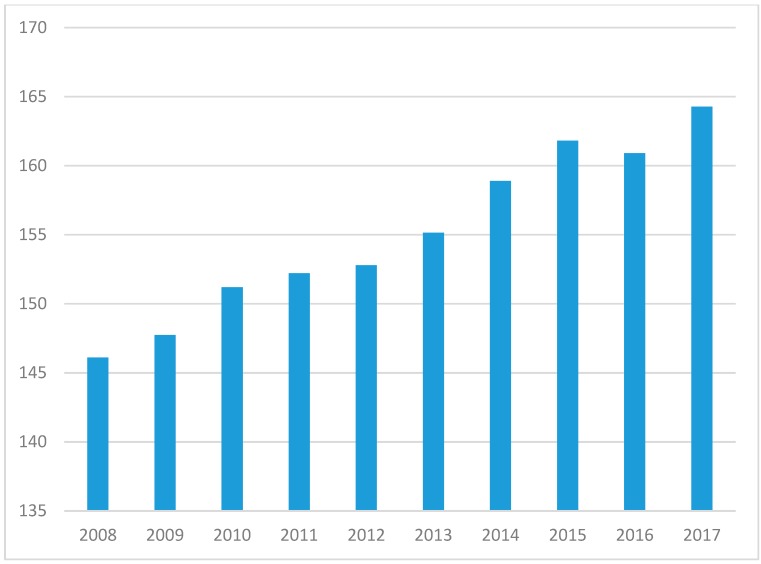
Ratio of older adults to pharmacist (2008 to 2017).

**Figure 4:**
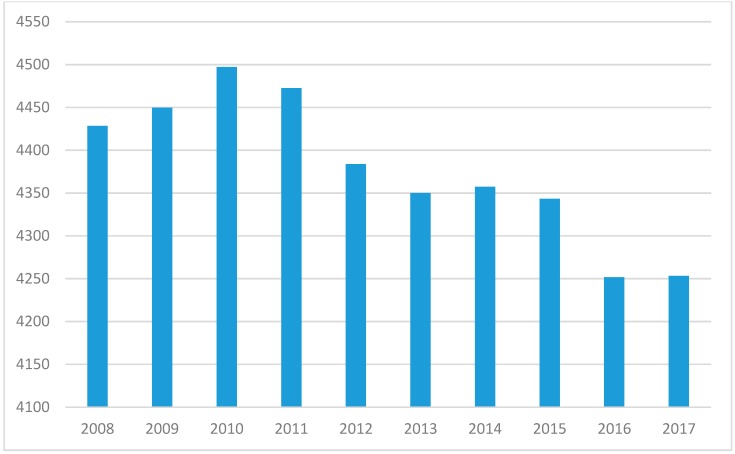
Ratio of medications to pharmacist (2008 to 2017).

On the surface, the shrinking number of people in the US per pharmacist suggests a glut of pharmacy practitioners and portends a reduction in salaries due to oversupply. However, a closer inspection of the data reveals potential indicators of a growing need for pharmacists. First, a rapidly aging US population has ignited usage of pharmaceuticals to an extent not experienced in history [11]. Second, US healthcare is in the middle of a rapid expansion in clinical, non-dispensing pharmacy services, coupled with an evolution of health care delivery to ambulatory care and community-based settings [3,12]. The largest pharmacy chain in the US, CVS Health, has begun touting its goal of providing health care services for chronic disease management at newly designed CVS pharmacies [13]. Given that CVS Health has acquired Aetna, one of the five largest health insurers in the US [14], it can rapidly begin to shape the health care delivery system by deciding covered services. These transformations are motivating, if not requiring, pharmacist positions to provide care. In contrast to the ratio of US persons per pharmacist ratio, the ratio of persons 65 years or older per pharmacist has increased from 146.1 older adults to each pharmacist in 2008 to 164.3 in 2017 (Figure 3). This represents a 12.4% increase in older adults to each pharmacist over that ten year span. Using the most recent MEPS data, people living in the US under 65 years averaged 3.15 unique prescription medications per year. People 65 years and over, averaged 8.85 unique prescription medications per year. Hence, an aging population translates to a rapid ascent of medication consumption. Factoring average prescription medication into the demographic shift reveals an intriguing trend. While the number of medications had been declining since 2010, with a drop in estimated prescription medications per pharmacist from 4,497.3 in 2008 to 4,251.7 in 2016, 2017 yielded an increase from the prior year to 4,253.2 (Figure 4). This trend is likely to continue given the acceleration in the aging of the US population. The US Census Bureau recently updated estimates due to the increase in aging rate of the population, in which the aged 65 years and older population will exceed the under 18 year old population by 2035 for the first time in history. By 2030, all of the baby boomer generation will be older than 65 years [1]. This portends an increase in total prescribed medications in the US with the concomitant need for more pharmacists to handle that enlarged volume. More importantly, the increasing necessity for pharmacist medication management will be needed because of the additional medications and the expanded population of seniors. Older adults are much more likely to experience an adverse drug event (ADE) than the younger population [15,16]. Consequently, the need to curate the medication regimen to reduce possible ADEs will grow. 

## 3. Implications

### 3.1. Economics and Policy

The increasing cost of medications will continue to pressurize a growing user population. Patients, payers, and providers are already struggling with rapidly increasing medication prices [17]. A recent report from the Kaiser Family Foundation found that 77% of respondents thought that prescription drug costs were unreasonable [18]. A recently published work that examined nationally representative data from 2010 to 2012 for 26 separate medication classes estimated there was $73 billion in excess spending due to usage of medications where a less expensive, but equally clinically effective medication was present [19]. The accompanying editorial recommended greater collaboration between pharmacists and physicians to improve usage of less costly, therapeutically equivalent medications [20]. From 2011 to 2015 the amount consumed by the ten highest cost medications increased from $21.5 billion to $28.4 billion in 2015 in US constant dollars, with an increasing presence of specialty medications. During the same period, the number of patients that received one of the ten highest cost medications dropped 32% from 12.9 million to 8.8 million. Medicare is spending a rapidly increasing amount of money on fewer older adults. At the current pace, Part D will pay approximately $40 billion for the ten highest cost medications by the end of 2020. Perhaps more importantly, the out-of-pocket spending by patients on their medications grew even more rapidly in that interval. The average cost per medication in the ten highest cost medications in 2015 US dollars grew 264% from $375 in 2011 to $1366 in 2015 [21]. A rapid expansion of the senior population colliding with a jump in expensive specialty medications will fuel the necessity of increasing clinical pharmacist engagement in helping patients manage the affordability of their medications while balancing clinical effectiveness. Further, the regulatory framework for direct-patient care services by pharmacists is increasingly well-positioned to deliver the pharmaceutical care that the evolving patient population deserves. In 38 states, pharmacists have provider status designations via the state code or a Centers for Medicare and Medicaid Services provision, with several other status enacting legislation to support pharmacist provider status [22]. Via creation of collaborative practice agreements with clinician prescribers, pharmacists can select, initiate, discontinue, and modify prescribing regimens [23]. This supports optimizing medication regimens in the framework of whole-person care that includes consideration of the cost concerns patients are now routinely grappling with. 

Stakeholder engagement by pharmacists and pharmacy organizations that fosters policy implementation to integrate clinical pharmacists in system-level patient care planning is also needed. As one example, participation by pharmacists in national dialogue on the opioid crisis led to policy recommendations advocating clinical pharmacy roles in harm-reduction and opioid use disorder treatment [24]. Discussion and dissemination of pharmacist activities that are salient to priority care goals are central to effective policy that supports improving patient and public health via clinical pharmacy. A recent nationally representative analysis by physician investigators found that more than 2.71 million older adults reported cost-related medication nonadherence. The researchers determined that 44.2% requested lower-cost medications from their provider, 11.5% used alternative therapies, and 5.3% purchased medications outside the US to reduce their out-of-pocket costs. The physician authors proposed medication therapeutic management as an important tool to improve adherence and outcomes for older adults struggling with their medications due to costs [25]. Coordinated policy making that integrates pharmacist clinical activities into achievement of system goals has never been more crucial. 

### 3.2. Research

Seismic shifts in care delivery, population age, medical economics, and pharmacy practice itself in the US requires advancing a research agenda that sheds light on the ability of the pharmacy profession to meet current and emerging clinical demands. Principally, research is needed to determine to what extent the current pharmacist supply is built for purpose in terms of mounting cognitive service demands. Even as automation unburdens pharmacists by assuming a greater role in dispensing, augmenting the advanced-practice skill set of pharmacists will remain a robust challenge. Research is needed to determine which areas of pharmacy practice and what specific skills will be demanded in the context of these shifts. The ability to “future-proof” the pharmacy profession to deliver on the promise of improved medicines and robust pharmaceutical care will benefit from funded research that characterizes best practices.

### 3.3. Education

As the pharmacy research paradigm evolves to elucidate the current state of practice and where modifications are needed, a critical by-product of that research is characterization of where pharmacy education must be focused or possibly created if none exists. Demographics have made manifest the importance of increasing pharmacists with geriatrics training, but emerging health technologies will also reveal where pharmacists must play a role. For instance, recent advances in dose-delivered mRNA therapies that could simulate the benefits of gene-therapy protein production without altering the patient’s genome are being hailed as breakthrough health technology [26,27]. Thus, creating precision medicine pharmacy programs that will educate students to be pharmacy experts in mRNA-based medications will need to be developed. Ensuring the necessary educational components are in place to meet evidence-driven pharmaceutical care goals is also required. The State of California has codified formal Advanced Pharmacist Practitioner designations from the California Board of Pharmacy [28]. However, ensuring that the necessary supply of expert clinical pharmacists and a robust system to provide uniform training that will quickly produce the number of advanced practice pharmacists needed in a state of roughly 40 million people is not guaranteed. Doctor of Pharmacy programs are now solidifying training on entrustable practice activities (EPAs) that include crafting a patient-centered care plan and providing longitudinal follow-up and evaluation [29]. It will be crucial to make sure that training for EPAs is tailored to the rapidly evolving delivery system and that cultivating a learning health care education system is the norm.

### 3.4. Practice

As this author has described in a prior publication, the importance of scaling comprehensive medication management (CMM) beyond a select group of integrated health systems and academic medical centers will be important to reach critical mass for nationwide population-level gains in clinical outcomes and reduction in global medical direct costs attributable to non-optimized medication regimens [2]. Importantly, increasing advanced practice pharmacy care is being rewarded by US federal mechanisms constructed to improve high-value care. Bipartisan legislation created the Medicare Access and Children’s Health Insurance Program Reauthorization Act of 2015 (MACRA) as an effort to achieve value-based payment reform. A new component of MACRA, the Merit Based Incentive Payments System (MIPS), which began enforcement in 2019, adjusts reimbursements to providers based on achievement of four categories of care: quality, promoting interoperability (formerly referred to as advancing care information), cost/resource use, and improvement activities. Each of these categories can be directly improved by pharmacists [30]. Quality measures include performance measures such as ensuring a complete and accurate medication record in the electronic health record. Promoting interoperability functions include sharing of the patient therapeutic plan with the patient and other clinicians to improve coordinated care. Cost/resource use improvement can be supported by pharmacists by identifying cost-effective medications and chronic disease management that reduces risk of hospitalization or emergency department visits. Improvement activities include expansion of medication therapeutic management programs and development of shared patient-clinician decision making that can be bolstered by the pharmacist. These activities also offer the ability to measure the impact of pharmacist care and quantify the influence on the clinic’s bottom line. Expanding clinical pharmacy services in diverse care settings and deliberate measurement of goals harmonizes perfectly with federal initiatives to improve value over volume care for patients. It is doubtless that the changing nature of medications and advancement of technology will impact pharmacist duties. Improvements in sustained-action delivery technology have helped patients reduce dosing frequency, improving adherence to medications. This development has led to reductions in the number of products dispensed by pharmacists. There are also an increasing number of specialty injectable and infusible medications that are being administered at a provider’s office without a pharmacy pick-up. Both of these factors present opportunities for growing pharmacy services. The potential reduction in filled prescriptions may afford the pharmacist more time for direct-patient care. The increasing array of costly, specialty medications has also stimulated increased interest in pharmacist administration of injected and infused medications. As these medications are increasingly managed at the pharmacy, payers are hoping to reduce costs attributed to the traditional buy-and-bill system of physician-administered drugs that has contributed to the increase in total health care costs [31]. 

This analysis and discussion are based on the most recent publically available, validated federal data on the pharmacist work force, US population, and prescription medication use data. Hence, the analysis and implications are subject to uncertainty, depending on future changes in these factors. Healthcare system-delivery shifts may also significantly impact the demand for pharmacists in unforeseen ways. This commentary paper has endeavored to incorporate potential changes based on current trends. Similar to a 2016 survey-based global analysis of the pharmacist labor supply, this analysis normalized the quantity of pharmacists based on country population size. However, the prior published global analysis did not incorporate average number of medications, change in age demographics of the population, and was subject to reporter and response bias regarding number of pharmacists per country [32]. 

## 4. Conclusions

It is clear that the increasing societal amount of total medication usage will necessitate an increase in clinical pharmacy services. As the burden of cost in the total health expenditure is increasingly being allocated to payment for medications, consideration will continue to grow on assessing pharmacy and medical benefits holistically as one budget. Manufacturers of innovative products are perpetuating the narrative that increased spending on medications will be more than offset by a reduction in medical spending. What will be necessary to meet this demand is an increase in supply of clinical pharmacists with advanced-practice training, equal to the task of working with patients and a care team, to optimize the medication regimen attuned to whole-person care. This includes review of the natural products, complementary, and alternative medications that patients are increasingly consuming. Growing the number of clinical pharmacists will also require expansion of the pool of qualified pharmacist educators that can train and inspire student pharmacists for these growing advanced patient-care roles. Increasing the number of post-PharmD residency and fellowship training programs is also desperately needed to ensure that student pharmacists passionate about advanced pharmacy practice are able to obtain the requisite training. Fulfilling the promise of modern pharmaceutical care to improve patients’ lives and reduce unnecessary health spending, against the backdrop of increasing medication use, will demand a commitment to innovative training and practice. The process has begun, but needs acceleration.
